# Alchemy and the New Age of Cardiac Muscle Cell Biology

**DOI:** 10.1371/journal.pbio.0030131

**Published:** 2005-04-12

**Authors:** Kenneth R Chien

## Abstract

Several studies have claimed to identify cardiac stem cells. But what criteria do such cells have to fulfil before we can be confident about their true potential?

*“Alchemy: a process of transforming something common into something special”—Webster's Dictionary*

A growing number of studies are reporting the isolation of cardiac stem cells from a variety of tissue sources and examining their effects on promoting the repair of the injured heart. In the current issue of *PLoS Biology*, the storyline takes an unexpected, interesting twist, as Neal Epstein and his colleagues report the isolation of a novel cell type from skeletal muscle that can adopt a highly differentiated cardiac muscle cell phenotype in vitro and in vivo [1].

In this study, the authors use a differential isolation procedure to remove the skeletal muscle cells and myoblasts (immature muscle cells), and then collect the cells that are negative for a cell-surface marker called Sca-1. Under defined in vitro conditions, these cells adopt a cardiomyocyte phenotype that goes beyond the simple expression of cardiac-restricted biochemical and molecular markers, extending to the types of single-cell physiological functions that are hallmarks of authentic cardiomyocytes, including action potentials, calcium transients, and contractile activity. They call the cells Spoc cells, an acronym for “skeletal-based precursor of cardiomyocytes.”

The study goes on to show that these cells can adopt this phenotype without the addition of cytokines or agents such as azacytidine that are known to activate the muscle gene program in nonmuscle cells. The cells also adopt the cardiac phenotype following their in vivo implantation into the ischemic heart following myocardial infarction, suggesting a potential therapeutic utility for these cells. Since Spoc cells were isolated from murine skeletal muscle, they may eventually allow the use of sophisticated conditional genetic tracking techniques to monitor the migration, maturation, and differentiation of the cells in the in vivo context.

Of course, a study with results this unexpected also raises a number of intriguing questions. Identifying the native location of Spoc cells, as well as the in vivo niche that insulates them from entering the differentiated cardiac program, will be valuable. A rigorous exploration of their developmental origin and their relationship to the other well-known cell types in skeletal muscle should be forthcoming. In this regard, a set of skeletal muscle stem cells, distinct from myoblasts, has also been found [2], and the question arises as to whether Spoc cells are related to these other skeletal muscle progenitors.

## Cardiomyocyte Precursors Abound

Other studies have reported the isolation of cells that can differentiate into cardiac muscle from diverse noncardiac tissues. In vitro and in vivo studies have suggested that these cells can adopt defined features of the cardiomyocyte phenotype when they are implanted into injured heart following myocardial infarction. It is possible that these studies are pointing to the existence of a rare circulating pool of precursor cells, which are able to home to the heart.

**Figure 1:**
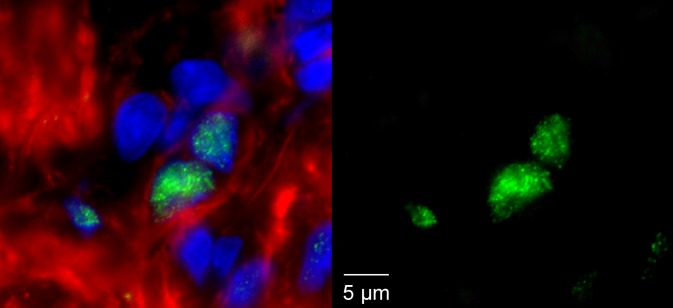
Islet-1-Positive Cardioblast in the Right Ventricular Chamber of the Neonatal Rat Heart Islet-1 nuclear staining depicted in green, nuclear staining in blue, myocyte marker staining in red. The left panel shows triple staining; the right panel shows single staining for Islet-1 expression.

To understand the provenance of these cells, it will become critical to identify a set of gene products, or markers, that are restricted to Spoc cells and to other potential progenitor cells. Since the protocols for the isolation of the Spoc cells have been developed in the mouse, it should also become possible to use state-of-the-art spatial and temporal control strategies for triggering irreversible lineage tracers in these cells in the in vivo context without isolating the cells per se. This will make it possible to follow the fate of these cells as they differentiate. Similar approaches have recently been used to identify a subset of rare, native cardiac progenitors (which are positive for the marker Islet-1) in the newborn hearts of mice, rats, and humans (Figure 1; [3]). Exploring the relationship of the Spoc cells to these native cardioblasts could reveal shared pathways that drive their formation, renewal, and differentiation.

## Defining a True Progenitor

One of the inherent caveats of the present study is the potential for phenotypic drift that occurs with prolonged growth of any cell type. It is possible, for example, that the genetic program of these cells gradually changes with time such that they can more easily adopt the cardiac phenotype. Heart and skeletal muscle share many key gene regulatory factors and downstream genetic programs, and the loss of a few key negative regulatory checkpoints might allow the cells to differentiate towards a cardiac phenotype. Again, documenting the existence of these cells in the in vivo context, particularly during stages of embryonic myogenesis, will be key.

There is already a clear precedent for this type of phenotypic drift between cardiac and skeletal muscle. H9c2 cells were originally derived from rat heart, but actually represent skeletal myoblasts, because they fuse and form myotubes following serum withdrawal. These cells express myogenic factors in the MyoD family, and do not express cardiac-restricted factors. Although the cells were initially isolated on the basis of their expression of cardiac markers, this is an example phenotypic drift—that is, the cells have drifted from a cardiac phenotype during repeated passage rather than simply representing skeletal muscle cells resident within the heart. Thus, the isolation of an immature cell from a heart does not necessarily denote that it is serving as a cardiac progenitor or stem cell. Potentially the same issue might hold for putative cardiac progenitor cells isolated from skeletal muscle, where the phenotype observed may not necessarily reflect its role in vivo within skeletal muscle.

One of the difficulties with alchemy and this new age of cardiac myocyte biology is related to defining a rigorous set of criteria that allow one to make the claim that the cell type of interest is truly a cardiac progenitor or stem cell and is acquiring a fully differentiated phenotype [4]. In this regard, the paper by Epstein and colleagues has done an admirable job of scoring for functional phenotypes (for example, having action potentials and calcium transients) that are far beyond the simple expression of cardiac muscle markers, which has been the phenotypic endpoint for many previous papers in the field. In addition, the authors go on to show the acquisition of the differentiated cardiac phenotype in the in vivo state in the absence of fusion with neighboring cardiac muscle cells, which has been a confounding variable in most other studies of this type. If a precursor cell simply fuses with a fully differentiated cell in a tissue, one could easily be fooled into thinking that the precursor has itself differentiated, as has been found for many types of putative cardiac stem cells [5]. Fortunately, techniques have been devised to detect such events.

In surveying the growing number of studies and claims of new cells that can acquire some type of heart cell phenotype in vitro or in vivo (for a review see [6]), it will become increasingly important to create a set of rigorous criteria that would distinguish between the following: authentic progenitor cells that are already committed to the cardiac lineage; pluripotent stem cells that can infrequently adopt the cardiac phenotype; phenotypic drift of other muscle progenitors with an increased propensity to enter cardiac lineages; and a variety of other cell types that can aberrantly express cardiac markers ectopically or by fusion with neighboring cardiac muscle cells.

Finding cell-type-specific markers for these cells will be critical, as opposed to generalized markers that do not allow the in vivo discrimination of their precise localization, mobilization, and differentiation in the intact muscle. The gold standard would then become the isolation of the cells from the intact organ after differentiation has occurred by creating genetically based or antibody-based approaches to identify and/or purify the already differentiated progeny from the intact muscle. Alternatively, new two-photon confocal microscopy approaches to identify the cells and then to monitor cardiac function in the intact heart should prove valuable [7].

For two decades, the bulk of our knowledge of molecular pathways that guide cardiac growth, development, and disease has been gleaned from a combination of in vivo studies in genetically engineered mice and primary cultures of neonatal and adult rat cardiomyocytes. Perhaps the most scientifically exciting aspect of this new age of cardiomyocyte biology is not simply related to cardiac repair. To date, there have been no continuous differentiated cardiac cell lines, a fact that has hampered the field for decades. The development of well-characterized cardiac progenitor cells offers the promise of using real genetic-based approaches to rapidly define the complex pathways that guide cardiac contractility, excitability, and lineage diversification into atrial, ventricular, and conduction system myocyte cell lineages.
